# Integrative pan-cancer analysis of UCP family and experimental validation identifies UCP2 as a potential therapeutic target for glioma

**DOI:** 10.3389/fcell.2025.1662654

**Published:** 2025-12-01

**Authors:** Wen Guo, Junru Chen, Shan Li, Xianghua Zeng, Xun Wu

**Affiliations:** 1 Department of Pharmacy, Affiliated Hospital of North Sichuan Medical College, Nanchong, Sichuan, China; 2 Department of Oncology, Mianyang Central Hospital, School of Medicine, University of Electronic Science and Technology of China, Mianyang, Sichuan, China; 3 The First School of Clinical Medicine, Lanzhou University, Lanzhou, Gansu, China; 4 Department of Oncology, Cancer Prevention and Treatment Institute of Chengdu, Chengdu Fifth People’s Hospital (The Second Clinical Medical College, Affiliated Fifth People’s Hospital of Chengdu University of Traditional Chinese Medicine), Chengdu, Sichuan, China

**Keywords:** bioinformatics, pan-cancer, glioma, UCP2, X-ray

## Abstract

**Objective:**

To evaluate the prognosis and therapeutic potential of the UCP family, particularly uncoupling protein 2 (UCP2), in 32 types of cancer through integrated analysis of TCGA and CGGA databases.

**Methods:**

Multi-omics data from TCGA, CGGA, GTEx, cBioPortal, and ROC Plotter were analyzed to assess UCP family expression patterns, prognostic significance, biological functions, immune cell infiltration, and genetic alterations across various cancers. *In vitro* experiments were carried out to assess UCP2’s impact on glioblastoma (GBM) aggressive traits and apoptosis.

**Results:**

UCP2 demonstrated significant overexpression in most malignancies, whereas other UCP family members showed reduced expression. High UCP2 expression is a prognostic risk factor for KIRP, LGG, and UVM, while it has protective effects in CESC, OV, SARC, and SKCM. Additional UCP members are associated with enhanced survival in certain cancers, such as BLCA and PAAD. Genetic analysis revealed negative regulation of UCP2 by DNA methylation. Functional enrichment linked the UCP family to epithelial-mesenchymal transition (EMT), G2M checkpoint, UV response, and mitotic processes across cancers. However, in more types of cancer, UCP2 is associated with immune-related pathways. Immune infiltration analysis revealed positive correlations between UCP family expression and stromal/immune scores but negative associations with immunosuppressive cells infiltration. Experimental validation in glioblastoma models confirmed that UCP2 knockdown attenuated EMT, impaired invasion, and improved radiosensitivity.

**Conclusion:**

This study establishes UCP2 as a prognostic indicator and potential therapeutic target for glioma.

## 1 Introduction

Gliomas, the most common and aggressive primary malignant brain tumors, present a formidable challenge in neuro-oncology due to their high heterogeneity, invasive growth, and resistance to traditional therapies (Liu et al., 2024). Even with standard treatment involving surgical resection and chemoradiotherapy, the prognosis for high-grade glioma patients remains dismal, with a median survival of only 12–15 months (Molinaro et al., 2019). The urgent need for novel molecular targets to disrupt glioma progression and improve therapeutic outcomes is highlighted by the limited efficacy of current treatments.

A key hallmark of glioma malignancy is metabolic reprogramming, through which tumor cells alter their energy metabolism to support rapid proliferation and survival within a nutrient-deprived microenvironment (Chai et al., 2024). Mitochondrial dysfunction and oxidative stress are pivotal to this process, fostering a dependency on adaptive mechanisms that protect tumor cells from apoptosis. The uncoupling protein (UCP) family, including UCP1-5, plays critical roles in mitochondrial metabolism by regulating proton leakage across the inner mitochondrial membrane and modulating the generation of endogenous reactive oxygen species (ROS) (Erlanson-Albertsson, 2003). While UCP1 is well-characterized in thermogenesis, UCP2 has garnered increasing attention in oncology due to its involvement in metabolic adaptation, redox balance, and therapy resistance (Brandi et al., 2016; Luby and Alves-Guerra, 2022). Previous studies indicate that UCP2 plays context-dependent roles in tumorigenesis across multiple cancer types, acting as either an oncogene or a tumor suppressor (Baffy, 2010; Esteves et al., 2014; Li et al., 2019). In gliomas, UCP2 overexpression has been linked to poor prognosis, suggesting its potential role in tumor progression (Wu et al., 2020; Vallejo et al., 2021). Mechanistically, UCP2 may promote glioma cell survival by enhancing glycolysis (the Warburg effect), mitigating oxidative stress, and conferring resistance to chemotherapy-induced apoptosis (Esteves et al., 2015; Wu et al., 2020). However, its functions in glioma radioresistance and epithelial-mesenchymal transition (EMT) remain poorly understood.

To evaluate the oncogenic potential of the UCP family, we conducted a thorough pan-cancer analysis using multi-omics data from TCGA and CGGA datasets. Our bioinformatic investigation identified UCP2 as a prominent prognostic marker within the UCP family, with high expression associated with unfavorable outcomes in various tumors, response to immunotherapy, and reduced efficacy of radiotherapy in glioma. Gene set enrichment analysis (GSEA) indicated that UCP2 expression positively correlates with EMT and immune modulation pathways, suggesting its dual role in promoting cancer cell invasion and regulating the tumor immune microenvironment. *In vitro* functional confirmed that UCP2 knockdown inhibits glioma cell migration, invasion, and EMT, and enhances cells sensitivity to X-ray irradiation.

In summary, our work provides a foundational framework for future research on UCP2-targeted strategies, highlighting its promise as a therapeutic target for glioblastoma (GBM).

## 2 Materials and methods

### 2.1 Data acquisition

Gene expression and phenotype data for pan-cancer analysis were obtained from the following sources: The Cancer Genome Atlas (TCGA) and GTEx data were downloaded from the UCSC Xena browser (https://xenabrowser.net/). The Xena resource provides harmonized RNA-seq data processed through a uniform computational pipeline (Toil), which minimizes batch effects and enables robust comparative analysis between TCGA tumors and GTEx normal tissues (Vivian et al., 2017; Goldman et al., 2020). The CGGA693 glioblastoma microarray dataset was obtained from the Chinese Glioma Genome Atlas (CGGA) database (http://www.cgga.org.cn/). This independent cohort was used for external validation.

For pan-cancer survival and GSEA, we utilized the standardized, batch-effect-adjusted datasets from TCGA Pan-Cancer Atlas consortium: The gene expression matrix (EBPlusPlusAdjustPANCAN_IlluminaHiSeq_RNASeqV2. geneExp.tsv) was generated by the PanCanAtlas team using the Firehose pipeline, which employs MapSplice for alignment and RSEM for transcript quantification. The data was normalized by setting the upper quartile to 1,000 for each sample. Clinical follow-up data and sample quality annotations were obtained from the same repository: https://gdc.cancer.gov/about-data/publications/pancanatlas. Samples flagged as poor quality in the annotation file were excluded from subsequent analysis. To enable cross-gene comparison within the TCGA cohort, the expression data for each gene were converted to a unit-free Z-score across tumor samples using the formula (x - μ)/σ, where x is the expression value, μ is the mean, and σ is the standard deviation. A full list of cancer types included in this study, along with their abbreviations as defined by TCGA, is provided in Supplementary Table S1.

### 2.2 Survival analysis

We conducted univariate Cox regression and Kaplan-Meier survival analysis to evaluate the prognostic significance of the UCP family in various cancer types. Forest plots were constructed using the R packages “survival” and “forestplot”. Kaplan-Meier survival curves, stratified by median gene expression levels, were generated with the R packages “survminer” and “survival”.

### 2.3 Genetic alteration analysis

The cBioPortal online database (http://cbioportal.org) was used to examine genomic alteration types and frequencies within TCGA pan-cancer atlas cohort. The Gene Set Cancer Analysis (GSCA) online platform was used to evaluate the single-nucleotide variations (SNV), copy number variations (CNV), and DNA methylation of the UCP family. For the DNA methylation analysis, only cancer types containing more than 10 matched tumor-normal sample pairs were included.

### 2.4 GSEA

The hallmark gene sets (h.all.v2023.2. Hs.symbols.gmt) were obtained from the Molecular Signatures Database (MSigDB; available at https://www.gsea-msigdb.org/gsea/index.jsp). Normalized enrichment scores (NES) and false discovery rates (FDR) across pan-cancer analyses were calculated using the “GSVA” and “clusterProfiler” R packages.

### 2.5 Immune cell infiltration analysis

Immune infiltration data for TCGA samples were obtained from the TIMER2.0 database (https://timer.cistrome.org/). The correlation between single-sample gene set enrichment analysis (ssGSEA) scores and immune infiltration levels across diverse cancer types was evaluated using the “GSVA” R package. The predictive capacity of UCP2 for immunotherapy response was assessed using ROC Plotter (https://rocplot.org/).

### 2.6 Cell culture and RNA interference

The U87 and U251 human glioma cell lines, obtained from the Institute of Modern Physics, Chinese Academy of Sciences (Lanzhou, China), were cultured under standard conditions and confirmed to be free of *mycoplasma* contamination. Cells were maintained in DMEM medium (HyClone, Logan, UT, United States) supplemented with 10% fetal bovine serum (FBS; ExCell Bio, Suzhou, Jiangsu, China) and 1% penicillin-streptomycin at 37 °C in a 5% CO2 atmosphere. Cells were transfected with UCP2-targeting siRNA (sc-42682) or a scrambled siRNA control (sc-37007) (both sourced from Santa Cruz Biotechnology, Heidelberg, Germany) and harvested 48–72 h later following the manufacturer’s guidelines. Transfection efficiency was assessed through quantitative real-time PCR and Western blot analysis.

### 2.7 Quantitative real-time polymerase chain reaction (qRT-PCR)

Total RNA was isolated from cultured cells using the GOONIE RNA extraction kit (Guangzhou, China, Cat. #400–100). Follow the manufacturer’s guidelines. Complementary DNA (cDNA) was synthesized using a commercial cDNA synthesis kit (Yeason, Shanghai, China). qRT-PCR was carried out using SYBR Green Master Mix (Yeason, Shanghai, China) on a Baiyuan real-time PCR detection system (Suzhou, China). The primer sequences used were as follows: UCP2, forward 5′-GTCCGGTTACAGATCCAAGGAG-3′ and reverse 5′-AGCCCATTGTAGAGGCTTCG-3′; β-actin, forward 5′-GACCACACCTACAATGAG-3′ and reverse 5′-GCATACCCCTCGTAGGG-3′. Relative mRNA expression levels were calculated using the 2^−ΔΔCT^ method, with β-actin as the internal reference gene.

### 2.8 Western blot

Cells were lysed using a mixture of RIPA buffer, PMSF, and phosphatase inhibitors (Solarbio, Beijing, China) at a ratio of 100:1:1. After incubation on ice for 30 min, the lysate was centrifuged at 12,000 r/min for 10 min at 4 °C. The supernatant containing the soluble cellular proteins was collected for subsequent analysis. Protein samples were separated by electrophoresis on 8% or 10% SDS-PAGE gels (Solarbio, Beijing, China) and then transferred to PVDF membranes (Millipore, Cork, Ireland). The membranes were blocked with 5% skim milk for 1 h at room temperature and subsequently incubated overnight with the following primary antibodies (all from Proteintech, Wuhan, China): anti-UCP2 (1:1,000), anti–Cleaved-Caspase-3 (1:1,000), anti–N-cadherin (1:2,000), anti–E-cadherin (1:10,000), anti-Vimentin (1:10,000), and anti–β-actin (1:200). After washing with TBST, the membranes were incubated for 1 h at room temperature with HRP-conjugated goat anti-rabbit IgG secondary antibody (Huabio, Hangzhou, China; 1:5,000). Protein bands were visualized using an enhanced chemiluminescence (ECL) detection reagent (Yeason, Shanghai, China) and quantified by grayscale analysis with ImageJ software.

### 2.9 X-ray irradiation

Irradiation was conducted with an X-Rad 225 system (Precision, North Branford, CT, United States) at 225 kV, 13.3 mA, and a dose rate of 2 Gy/min.

### 2.10 Wound healing assay and transwell invasion assay

Cells were cultured in 6-well plates until they reached 90%–100% confluence; next, a scratch was made in the cell layer with a sterile 200 μL pipette tip, then a serum-free DMEM medium (HyClone, Logan, UT, United States) was added. Wound closure was examined at 0 and 24 h using a ×40 magnification inverted microscope (Olympus, Japan). The migration rate was calculated by measuring the change in wound width over time using ImageJ software (NIH, United States).

Cell invasion was evaluated using Transwell chambers with 8 μm pores (Corning, United States). Matrigel (BD Biosciences, United States) was used to pre-coat the membranes. 20,000 cells in serum-free DMEM were placed in the upper chamber, with the lower chamber containing complete medium with 10% FBS (ExCell Bio, Suzhou, Jiangsu, China). After 24 h of incubation, the cells that penetrated Matrigel and reached the lower surface were fixed, stained, and photographed using a microscope (Olympus, Japan) with a magnification of 200×.

### 2.11 Statistical analysis

The R software (version 4.3.1) and RStudio (2023.09.0) were used for bioinformatics analysis, while experimental data were analyzed with GraphPad Prism (version 10.1.2; San Diego, CA, United States). Continuous variables following a normal distribution are presented as mean ± standard deviation (SD), and comparisons between groups were performed using Student’s t-test. Otherwise, the Mann–Whitney U test was applied. Survival analyses were performed using univariate Cox proportional hazards regression, Kaplan–Meier estimation, and log-rank tests. Associations with immune cell infiltration were assessed using Spearman’s rank correlation coefficients. All statistical tests were two-tailed, with significance levels defined as follows: *P < 0.05, **P < 0.01, ***P < 0.001, and ****P < 0.0001.

## 3 Results

### 3.1 UCP family expression in normal and cancer tissues

**FIGURE 1:**
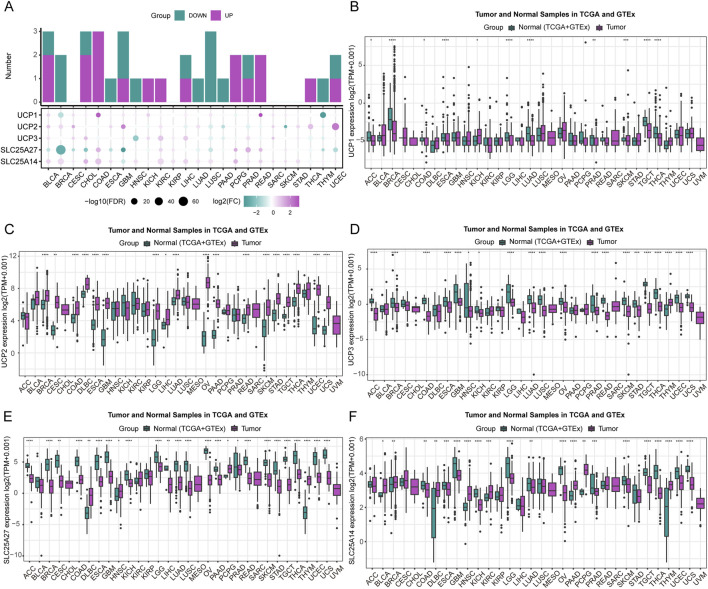
Expression of UCP family members. **(A)** A composite stacked bar and bubble plot showing the differential expression of UCP genes at the pan-cancer level. **(B–F)** Expression of individual UCP genes across various cancer types, analyzed using data from the TCGA and GTEx databases.

We comprehensively analyzed UCP family gene expression patterns across various human cancers using pan-cancer data from The Cancer Genome Atlas (TCGA). As illustrated in Figure 1A, the five UCP family genes exhibited distinct expression profiles across multiple cancer types. Significant upregulation was noted in BLCA, COAD, CHOL, KICH, KIRC, LIHC, PCPG, PRAD, READ, THCA, and UCEC, while downregulation occurred in BRCA, ESCA, GBM, HNSC, LUAD, LUSC, PAAD, and THYM. We compared gene expression between tumor and normal tissues by integrating data from the TCGA and GTEx databases. UCP1, UCP3, SLC25A27, and SLC25A14 showed significantly lower expression in most tumor tissues (Figures 1B–E). In contrast, UCP2 was markedly upregulated across the majority of cancers analyzed (Figure 1F). These consistent expression patterns suggest that UCP2 may function as an oncogene in diverse cancer contexts, while UCP1, UCP3, SLC25A27, and SLC25A14 are potentially involved in tumor-suppressive mechanisms.

### 3.2 Survival analysis

**FIGURE 2:**
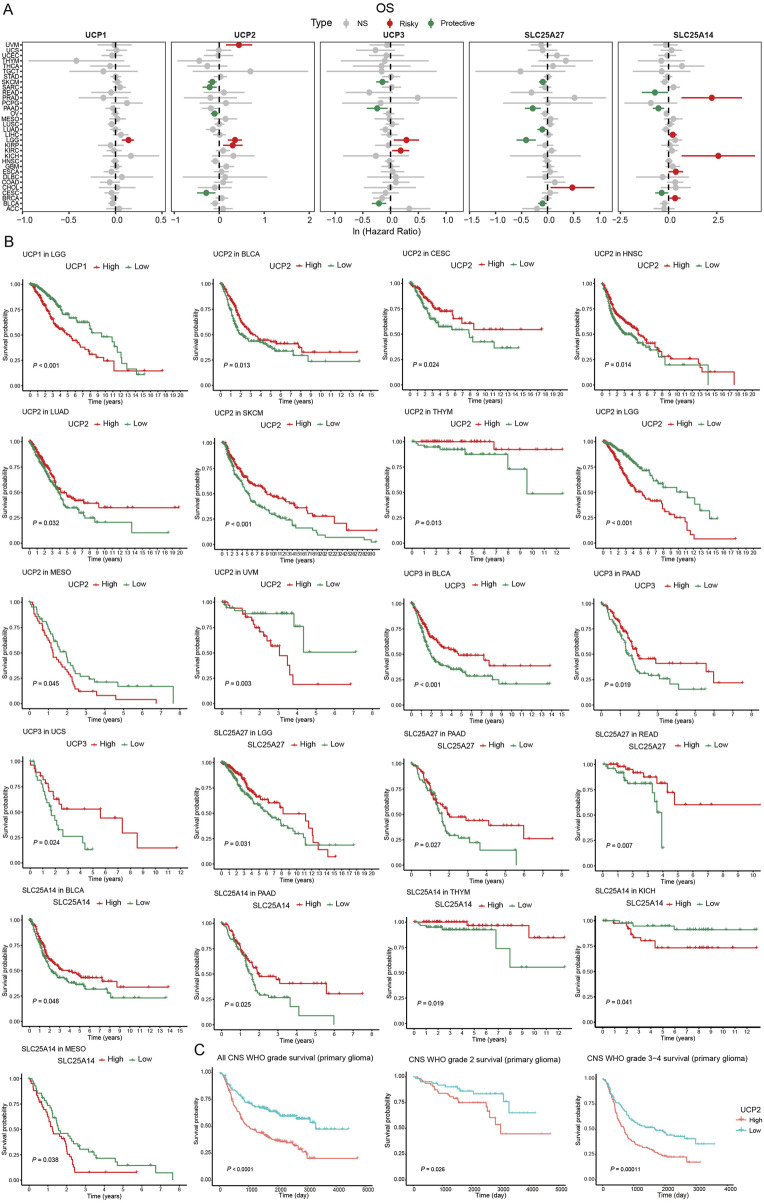
Survival analysis of UCP family based on TCGA and CGGA databases. **(A)** Univariable Cox regression analysis of UCP family gene expression for OS based on TCGA database. **(B)** Kaplan-Meier curves of OS of UCP family genes based on TCGA database. **(C)** Kaplan-Meier curves of OS for UCP2 expression in glioma patients based on the CGGA database.

To assess the prognostic significance of UCP family genes, we conducted univariate Cox regression and Kaplan–Meier analyses to assess their associations with overall survival (OS), disease-specific survival (DSS), disease-free interval (DFI), and progression-free interval (PFI) across various cancers (Figures 2A,B; Additional file: Supplementary Figure S1). UCP1 was a significant risk factor in LGG, where high expression correlated with reduced OS. UCP2 exhibited context-dependent roles: it functioned as a risk factor in KIRP, LGG, and UVM, while it was associated with improved outcomes in CESC, OV, SARC, and SKCM. Elevated UCP2 expression was correlated with prolonged OS in BLCA, CESC, HNSC, LUAD, SKCM, and THYM, whereas it predicted poorer OS in LGG, MESO, and UVM. UCP3 served as a risk factor in KIRC and LGG, but as a protective factor in BLCA, PAAD, and SKCM. Higher UCP3 expression was associated with improved OS in BLCA, PAAD, and UCS. SLC25A27 was identified as a risk factor in CHOL but a protective factor in BLCA, LGG, LUAD, PAAD, and SKCM. Elevated SLC25A27 levels correlated with better OS in LGG, PAAD, and READ. SLC25A14 acted as a risk factor in BRCA, ESCA, KICH, LIHC, and PRAD, but was a protective risk in CESC, PAAD, and READ. High SLC25A14 expression was linked to improved OS in BLCA, PAAD, and THYM, and reduced OS in KICH and MESO. To further validate these findings in glioma, we analyzed data from the CGGA database. Consistent with TCGA results, elevated UCP2 expression correlated significantly with poorer prognosis in glioma patients (CNS WHO grades 2–4) (Figure 2C).

**FIGURE 3:**
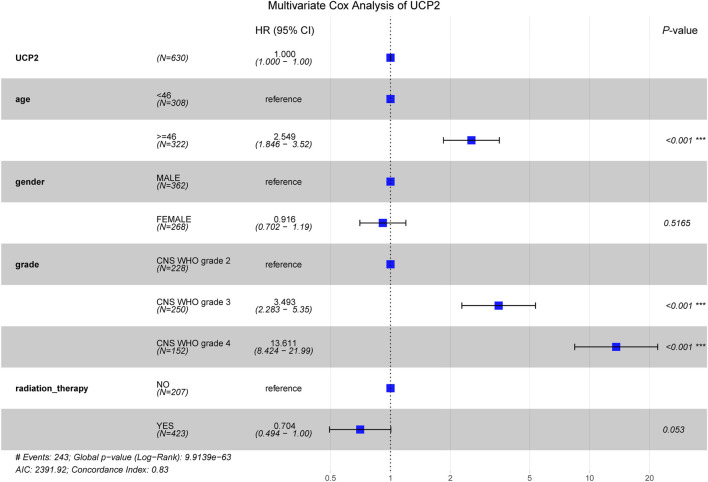
Multivariate COX analysis of UCP2 based on TCGA database glioma cohort.

Multivariate Cox analysis showed that advanced age (≥46 years) and high-grade glioma (CNS WHO grades 3–4) were significantly associated with poorer OS in glioma patients, while radiation therapy emerged as a favorable prognostic factor correlated with enhanced survival (Figure 3).

### 3.3 Genetic alteration analysis

**FIGURE 4:**
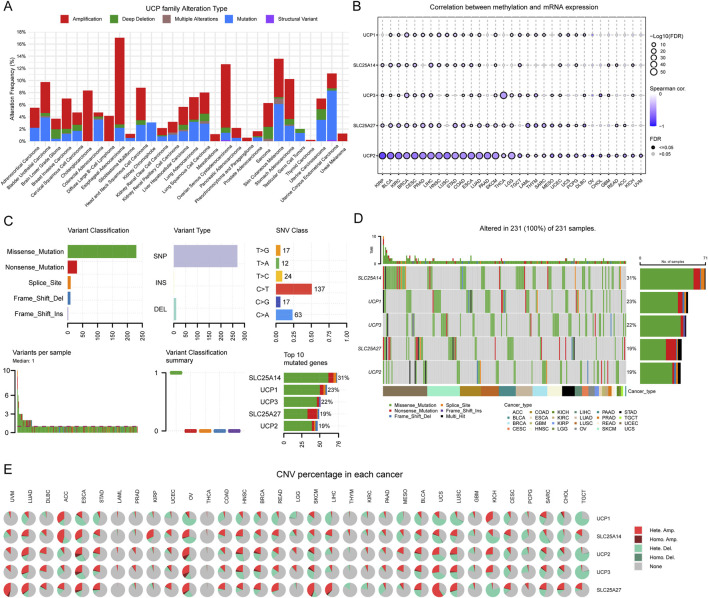
Genetic variation analysis of UCP family. **(A)** Total frequency and genetic variations of UCP family according to the cBioPortal online platform—pan-cancer analysis based on TCGA pan-cancer atlas. **(B)** Correlation between DNA methylation and UCP family mRNA expression in pan-cancer according to the GSCA online platform. **(C)** The SNV summary of UCP family in different types of cancers. **(D)** Waterfall plot of the mutation frequencies of the UCP family in pan-cancer. **(E)** The percentage of heterozygous and homozygous CNV profiles of UCP family in pan-cancer.

We comprehensively analyzed genetic alterations—including mutation types, DNA methylation, single-nucleotide variants (SNV), and copy number variations (CNV)—across multiple cancers to investigate the potential mechanisms underlying aberrant expression of UCP family genes. The UCP gene family displayed diverse genomic alterations, such as amplification, mutation, deep deletion, and structural variants, prevalent in numerous cancer types (Supplementary Figure S2). Across the family, amplification and point mutations emerged as the most frequent genetic changes (Figure 4A). DNA methylation analysis revealed a general inverse correlation with mRNA expression levels of UCP genes, a trend particularly pronounced for UCP2 in the pan-cancer context (Figure 4B). Further examination of SNV and CNV patterns showed that missense mutations represent the predominant SNV class in the UCP family, followed by nonsense mutations, with single-nucleotide polymorphisms (SNPs) constituting the majority of all variants (Figure 4C). The most common nucleotide changes involved C-to-T and G-to-T conversions. Among the family members, SLC25A14 exhibited the highest mutation frequency (31%), followed by UCP1 (23%), UCP3 (22%), SLC25A27 (19%), and UCP2 (19%) (Figure 4D). CNV profiles were characterized primarily by heterozygous amplification and deletion, along with instances of homozygous amplification (Figure 4E).

### 3.4 GSEA

**FIGURE 5:**
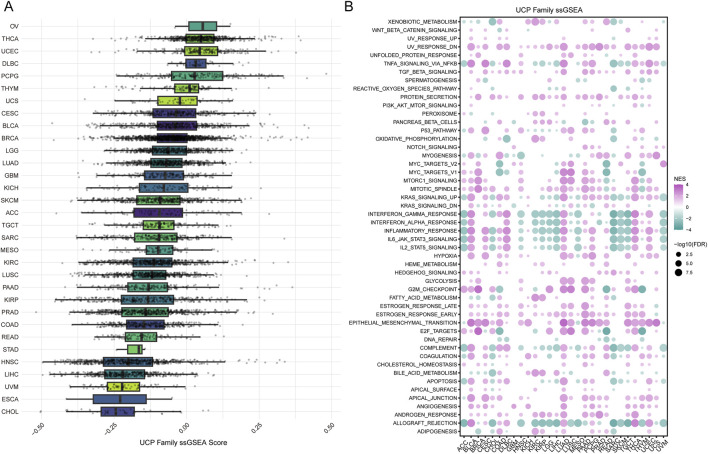
Analysis of UCP family activity and its association with hallmark pathways. **(A)** Distribution of ssGSEA scores quantifying UCP family activity across various cancer types. **(B)** Correlations between UCP family ssGSEA scores and the enrichment scores of hallmark cancer pathways in the pan-cancer cohort.

We conducted Gene Set Variation Analysis (GSVA) to compute single-sample Gene Set Enrichment Analysis (ssGSEA) scores for the UCP family and performed subsequent enrichment analysis. The ssGSEA scores of the UCP family showed limited variation across cancer types, with ovarian cancer (OV) exhibiting the highest score. Most cancer types—except OV, THCA, UCEC, DLBC, PCPG, and THYM—displayed ssGSEA scores below zero (Figure 5A). Enrichment analysis based on 50 hallmark gene sets revealed significant associations between the UCP family and EMT, the G2M checkpoint, UV response, and mitotic processes in the majority of cancers. Additionally, the UCP family was linked to immune regulatory responses in BLCA, DLBC, LUAD, THCA, and UCEC (Figure 5B). Notably, UCP2 was not only involved in EMT and NF-κB signaling pathways across multiple malignancies but also correlated with numerous immune-related pathways in a wide range of cancer types (Supplementary Figure S3).

### 3.5 Immune infiltration analysis

**FIGURE 6:**
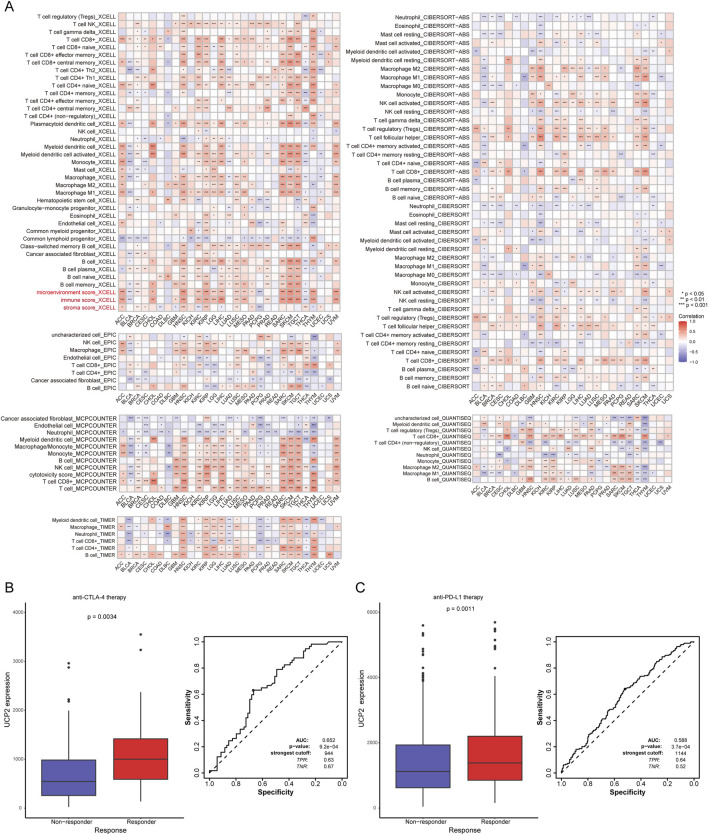
The UCP family in tumor immunity and immunotherapy response. **(A)** Correlations of UCP family activity (ssGSEA score) with immune cell infiltration levels in pan-cancer. **(B)** ROC curve analyzing the utility of UCP2 expression in predicting response to anti-CTLA-4 therapy. **(C)** ROC curve analyzing the utility of UCP2 expression in predicting response to anti-PD-L1 therapy.

We applied multiple computational algorithms to systematically evaluate the relationship between the UCP family and tumor-infiltrating immune cells. As shown in Figure 6A, UCP family activity scores were significantly positively correlated with stromal, immune, and microenvironmental scores in most cancer types. These findings were consistently supported by pan-cancer immune cell heatmaps generated through diverse algorithmic approaches. Conversely, the UCP family exhibited significant negative correlations with the infiltration levels of cancer-associated fibroblasts (CAFs), neutrophils, and endothelial cells across most malignancies. Using the ROC Plotter database, we further assessed the predictive significance of UCP2 levels in response to immunotherapy. These results indicated that patients who responded to anti-CTLA-4 and anti-PD-L1 therapies had significantly higher UCP2 expression compared to non-responders (Figures 6B,C). These findings indicate that UCP2 expression may serve as a predictive biomarker for the efficacy of immune checkpoint inhibitor treatment.

### 3.6 UCP2 knockdown inhibited GBM cell migration, invasion and EMT

**FIGURE 7:**
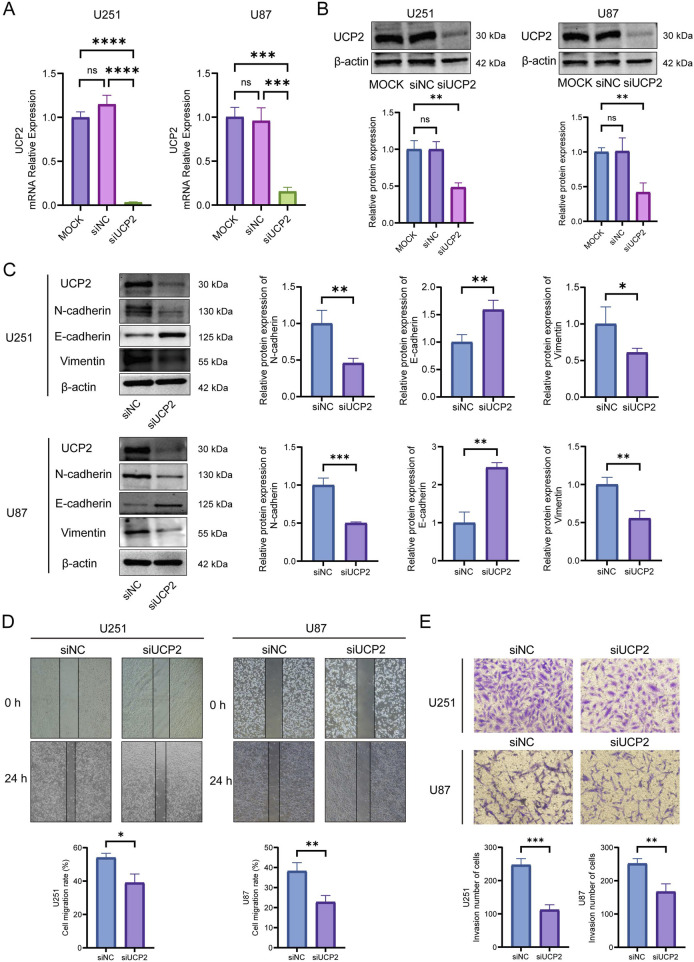
UCP2 knockdown suppresses the migration, invasion, and EMT of GBM cell lines. UCP2 knockdown efficiency verification by **(A)** qRT-PCR and **(B)** Western blot. **(C)** Western blot assay detected the changes in EMT-related proteins. The changes in cell migration and invasion capacity after UCP2 knockdown were detected by **(D)** wound healing assay and **(E)** Transwell invasion assay.

We used U251 and U87 GBM cell lines to experimentally validate crucial bioinformatics predictions. Using siRNA-mediated knockdown, we successfully reduced UCP2 expression in both U251 and U87 cells, as confirmed at the mRNA and protein levels (Figures 7A,B). Given GSEA results suggesting UCP2’s involvement in EMT, we examined the impact of UCP2 knockdown on EMT marker expression in GBM cells. Western blot results demonstrated that silencing UCP2 significantly reduced N-cadherin and vimentin protein while enhancing E-cadherin levels in both cell lines (Figure 7C). Considering the known association between EMT and metastasis, we subsequently assessed the role of UCP2 in cellular migration and invasion. Wound healing assays demonstrated that silencing UCP2 markedly impaired the migration of both U251 and U87 cell lines (Figure 7D). Consistent with this, transwell invasion assays revealed a significant reduction in invasive ability upon UCP2 suppression (Figure 7E).

### 3.7 UCP2 knockdown increased GBM cell apoptosis induced by irradiation

**FIGURE 8:**
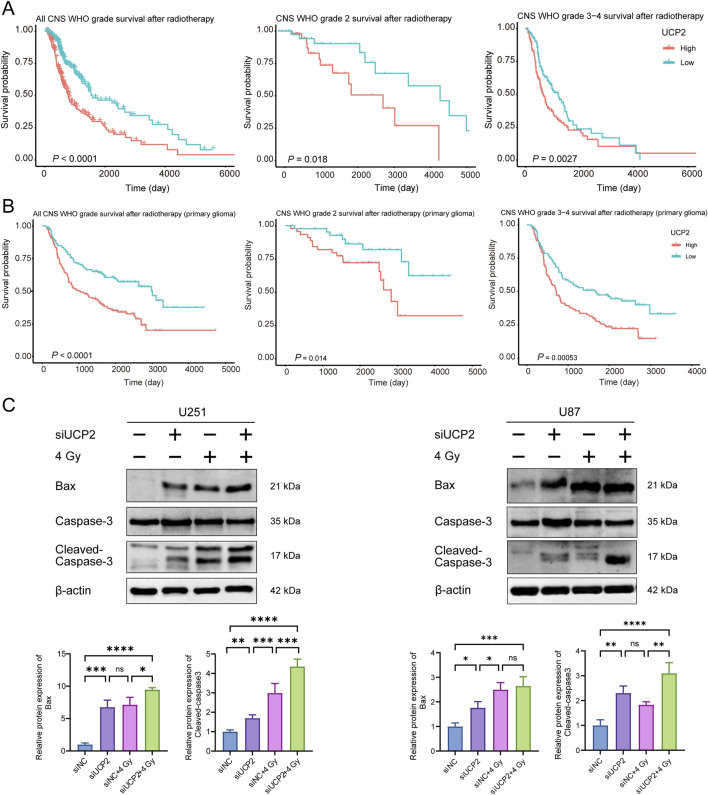
Knockdown of UCP2 increased cell apoptosis induced by irradiation. Kaplan-Meier curves of glioma patients with different UCP2 expression levels in **(A)** TCGA and **(B)** CGGA databases after radiotherapy. **(C)** Western blot detection of apoptosis-related proteins after UCP2 knockdown and/or irradiation.

To investigate the association between UCP2 expression and radiotherapy response, GBM patients who received radiotherapy were selected from the TCGA and CGGA cohorts and divided into high- and low-UCP2 expression groups according to median mRNA levels. The Kaplan–Meier curves indicated that high UCP2 expression was associated with significantly poorer overall survival among irradiated patients compared to those with low UCP2 expression (Figures 8A,B).

To examine whether UCP2 knockdown promotes apoptosis and enhances radiosensitivity in GBM, we evaluated apoptosis-related protein expression via Western blotting in U251 and U87 cells. The results demonstrated that UCP2 knockdown not only increased baseline apoptosis in both cell lines but also significantly enhanced radiation-induced apoptosis following X-ray irradiation (Figure 8C).

## 4 Discussion

By integrating multi-omics data across diverse cancer types, pan-cancer analysis enables the systematic evaluation of candidate genes for their prognostic relevance and potential as predictive biomarkers in malignant tumors (Chen et al., 2025b). This study integrates bioinformatics and experimental validation to perform a comprehensive pan-cancer analysis of the UCP family, emphasizing UCP2’s role in glioma malignancy and treatment response. Our findings indicate that UCP2 is often overexpressed in various cancer types, correlating with poor prognosis and aggressive tumor characteristics. Furthermore, UCP2 expression was associated with radiation resistance in glioma. Functional experiments demonstrated that UCP2 knockdown inhibits EMT, migration, and invasion in glioblastoma cells, while increasing apoptosis induced by irradiation. These *in vitro* results suggest that UCP2 could serve as a potentially therapeutic target and a prognostic biomarker in glioma patients, warranting further investigation.

Our pan-cancer analysis revealed distinct expression patterns among UCP family members across different tumor types. While UCP1, UCP3, SLC25A27, and SLC25A14 were frequently downregulated in most cancers, UCP2 showed consistent upregulation, indicating divergent functional roles in oncogenesis. The elevated expression of UCP2 in multiple malignancies, including glioblastoma, suggests its potential involvement in tumorigenesis and progression. This finding is consistent with previous reports implicating UCP2 in metabolic reprogramming—an established hallmark of cancer (Esteves et al., 2014; Esteves et al., 2015; Beikbaghban et al., 2024). UCP2-mediated mitochondrial uncoupling is associated with reduced ROS generation, which may enhance tumor cell survival under oxidative stress conditions. (Mailloux and Harper, 2011). Interestingly, although ROS levels are typically moderately elevated in tumor cells (Wu et al., 2024), UCP2 appears to play an essential role in maintaining redox homeostasis and supporting cancer cell viability. Conversely, the frequent downregulation of other UCP family members, such as UCP3 and SLC25A27, across tumors suggests their potential roles as tumor suppressors, although this remains speculative without functional validation. Consistent with this, survival analysis indicated that higher expression of these genes is correlated with improved clinical outcomes in certain cancers, highlighting the need for further mechanistic studies into their protective functions.

UCP2, a mitochondrial uncoupling protein, is crucial for energy metabolism and oxidative stress regulation (Pitt, 2015). This study revealed that elevated UCP2 expression was associated with improved prognosis in cancers such as BLCA and CESC, whereas it correlated with poorer outcomes in LGG, MESO, and UVM. This tissue-specific duality in prognostic significance could be explained by contextual factors such as the tumor microenvironment (TME) and activation of distinct molecular pathways. These findings offer fresh perspectives on the biological complexity and diversity of cancer.

DNA methylation analysis is a critical biomarker for the diagnosis and classification of gliomas, enabling the stratification of patients into molecular subgroups with distinct clinical outcomes, thereby providing key insights for prognostic evaluation and treatment strategy formulation (Capper et al., 2018). Genetic and epigenetic analyses in this study demonstrate that DNA methylation acts as a key regulatory mechanism for UCP2 expression in various cancers, including glioma. A significant negative correlation was observed between the methylation level of the UCP2 promoter region and its mRNA expression. Cellular experiments further revealed that UCP2 mediates radiotherapeutic sensitivity in glioma cells, and inhibition of UCP2 expression enhances radiation-induced cell death. These findings underscore the importance of DNA methylation as a factor to consider in glioma treatment strategies, given that our data raise the possibility that targeting DNA methylation might influence glioma radiosensitivity in part through the regulation of UCP2 expression.

Given the aggressive behavior and therapy-resistant nature of glioblastoma (Stupp et al., 2014), we sought to explore potential mechanisms. One potential explanation for the association between UCP2 and poor clinical outcomes observed in bioinformatics analysis is its role in promoting EMT, as supported by our *in vitro* findings of EMT protein level changes upon UCP2 knockdown. Our experiments showed that knocking down UCP2 in GBM cells disrupts the levels of EMT related proteins, indicating EMT suppression. Since EMT is known to facilitate tumor dissemination and confer therapy resistance (Liu et al., 2019), targeting UCP2 may represent a promising strategy for inhibiting these aggressive traits.

UCP2 knockdown markedly reduced GBM cell motility and invasion, as evidenced by wound healing and transwell invasion assays. These results align with previous studies that have implicated UCP2 in cytoskeletal reorganization and the promotion of metastatic behavior in various cancer types (Wang et al., 2020; Du et al., 2023). UCP2 may modulate mitochondrial dynamics and redox homeostasis, influencing cell motility and extracellular matrix interactions (Caggiano and Taniguchi, 2024).

A critical finding of this study is the role of UCP2 in modulating radiation response. Patients with high UCP2 expression exhibited worse survival outcomes following radiotherapy, suggesting that UCP2 may be involved in the radiation resistance of glioma cells. Radiation therapy exerts cytotoxic effects partly through ROS generation, and UCP2’s mitochondrial uncoupling activity can reduce ROS accumulation (Echtay et al., 2002; Chen et al., 2025a). Thus, we hypothesize that UCP2 overexpression could protect tumor cells from radiation-induced cell death by mitigating ROS accumulation. Our Western blot data showing increased apoptosis upon UCP2 depletion support this hypothesis. UCP2 knockdown enhanced radiation-induced apoptosis in GBM cells, implying that UCP2 inhibition could sensitize tumors to radiotherapy. This is particularly relevant given that radiotherapy remains a cornerstone of glioblastoma treatment, yet resistance remains a major clinical challenge (Ali et al., 2020). Next studies should explore whether combining UCP2 inhibition with radiotherapy improves therapeutic efficacy in preclinical glioma models.

Immune infiltration analysis showed that UCP family gene expression positively correlates with stromal and immune scores across multiple cancer types. Notably, tumors with high UCP family scores showed reduced infiltration of immunosuppressive cells such as CAFs. Our previous bioinformatics analysis indicated a positive association between elevated NOX4 expression–a key generator of endogenous ROS–and increased CAFs abundance (Wu et al., 2025). These results suggest that redox regulation may have a critical impact on the tumor immune microenvironment.

One study showed that targeting the UCP2 pathway can overcome resistance to programmed cell death protein-1 blockade (Cheng et al., 2019). Consistently, we observed higher UCP2 expression in patients who responded to anti-CTLA-4 and anti-PD-L1 treatments, supporting its potential role as a predictive biomarker for immune checkpoint inhibitor efficacy. The apparent contradiction between its immunosuppressive and immune-favorable roles may reflect context-dependent immunomodulatory functions of UCP2. Further investigation is necessary to elucidate the precise mechanisms through which UCP2 influences antitumor immunity.

Our integrated analysis and *in vitro* functional data highlight UCP2 as a potential multifaceted oncogenic factor with prognostic and therapeutic relevance in glioma. Its frequent overexpression in aggressive tumors and contribution to therapy resistance support its further investigation as a promising therapeutic target. Small-molecule inhibitors of UCP2, such as genipin, have demonstrated preclinical efficacy in other cancer types (Dando et al., 2017), supporting further evaluation in glioblastoma models.

Furthermore, the association between UCP2 and EMT suggests that its inhibition may attenuate metastatic dissemination—a primary cause of treatment failure in solid tumors. Combining UCP2-targeted agents with conventional radiotherapy or immunotherapy could yield synergistic therapeutic effects, especially in UCP2-high glioma subtypes. Subsequent research should focus on validating these combination strategies *in vivo* and developing robust biomarkers for patient stratification.

The study is subject to several limitations. The identification of pathogenic genes was based on bulk RNA-seq data from the TCGA database. Without single-cell RNA sequencing (scRNA-seq) data, it is challenging to accurately identify the specific cellular subpopulations, such as glioma stem cells, malignant astrocytes, or tumor-associated macrophages, where UCP2 is highly expressed. This limitation hinders understanding of its cell type-specific functional roles (Xu et al., 2025). Additionally, this study did not utilize machine learning algorithms like LASSO regression, random forest, or support vector machine (SVM) for integrative multi-omics analysis, which might improve the predictive model’s robustness and the reliability of gene screening (Xu et al., 2022).

## 5 Conclusion

This integrative research identifies UCP2 as a potential key regulator of glioma progression, EMT, and radiation resistance in preclinical models. Our findings, primarily derived from bioinformatics and *in vitro* studies, underscore UCP2 as a candidate therapeutic target and highlight novel avenues for future research to improve glioblastoma treatment. Further preclinical and clinical investigations are warranted to translate these insights into therapeutic applications.

## Data Availability

The datasets generated and analyzed during the current study are available from the corresponding author on reasonable request.
