# Supplementary material for: Integrative pan-cancer analysis of UCP family and experimental validation identifies UCP2 as a potential therapeutic target for glioma
Source: Front Cell Dev Biol. 2025 Dec 1;13:1662654. doi: 10.3389/fcell.2025.1662654 (PMC12702733; doi:10.3389/fcell.2025.1662654)
Supplement: Supplementary file 1 [file DataSheet1.docx]

**Table S1** Full names and abbreviations of the 33 cancer types in TCGA.

| Serial number | Cancer Types | Abbreviations |
| --- | --- | --- |
| 1 | Adrenocortical carcinoma | ACC |
| 2 | Bladder Urothelial Carcinoma | BLCA |
| 3 | Breast invasive carcinoma | BRCA |
| 4 | Cervical squamous cell carcinoma and endocervical adenocarcinoma | CESC |
| 5 | Cholangiocarcinoma | CHOL |
| 6 | Colon adenocarcinoma | COAD |
| 7 | Lymphoid Neoplasm Diffuse Large B-cell Lymphoma | DLBC |
| 8 | Esophageal carcinoma | ESCA |
| 9 | Glioblastoma multiforme | GBM |
| 10 | Head and Neck squamous cell carcinoma | HNSC |
| 11 | Kidney Chromophobe | KICH |
| 12 | Kidney renal clear cell carcinoma | KIRC |
| 13 | Kidney renal papillary cell carcinoma | KIRP |
| 14 | Acute Myeloid Leukemia | LAML |
| 15 | Brain Lower Grade Glioma | LGG |
| 16 | Liver hepatocellular carcinoma | LIHC |
| 17 | Lung adenocarcinoma | LUAD |
| 18 | Lung squamous cell carcinoma | LUSC |
| 19 | Mesothelioma | MESO |
| 20 | Ovarian serous cystadenocarcinoma | OV |
| 21 | Pancreatic adenocarcinoma | PAAD |
| 22 | Pheochromocytoma and Paraganglioma | PCPG |
| 23 | Prostate adenocarcinoma | PRAD |
| 24 | Rectum adenocarcinoma | READ |
| 25 | Sarcoma | SARC |
| 26 | Skin Cutaneous Melanoma | SKCM |
| 27 | Stomach adenocarcinoma | STAD |
| 28 | Testicular Germ Cell Tumors | TGCT |
| 29 | Thyroid carcinoma | THCA |
| 30 | Thymoma | THYM |
| 31 | Uterine Corpus Endometrial Carcinoma | UCEC |
| 32 | Uterine Carcinosarcoma | UCS |
| 33 | Uveal Melanoma | UVM |


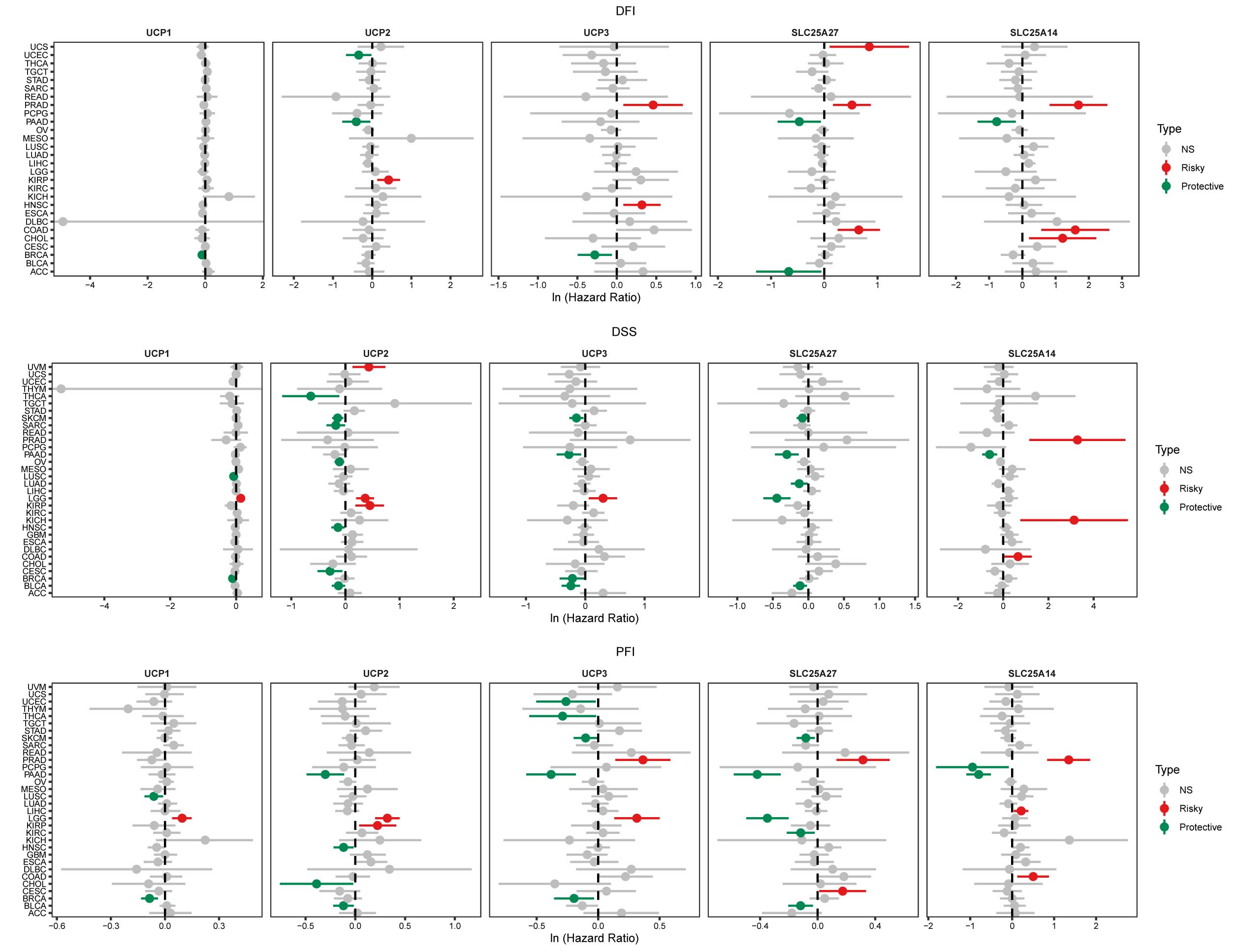


**FIGURE S1**

Univariable Cox regression analysis for DFI, DSS and PFI of UCP family genes expression in pan-cancer.


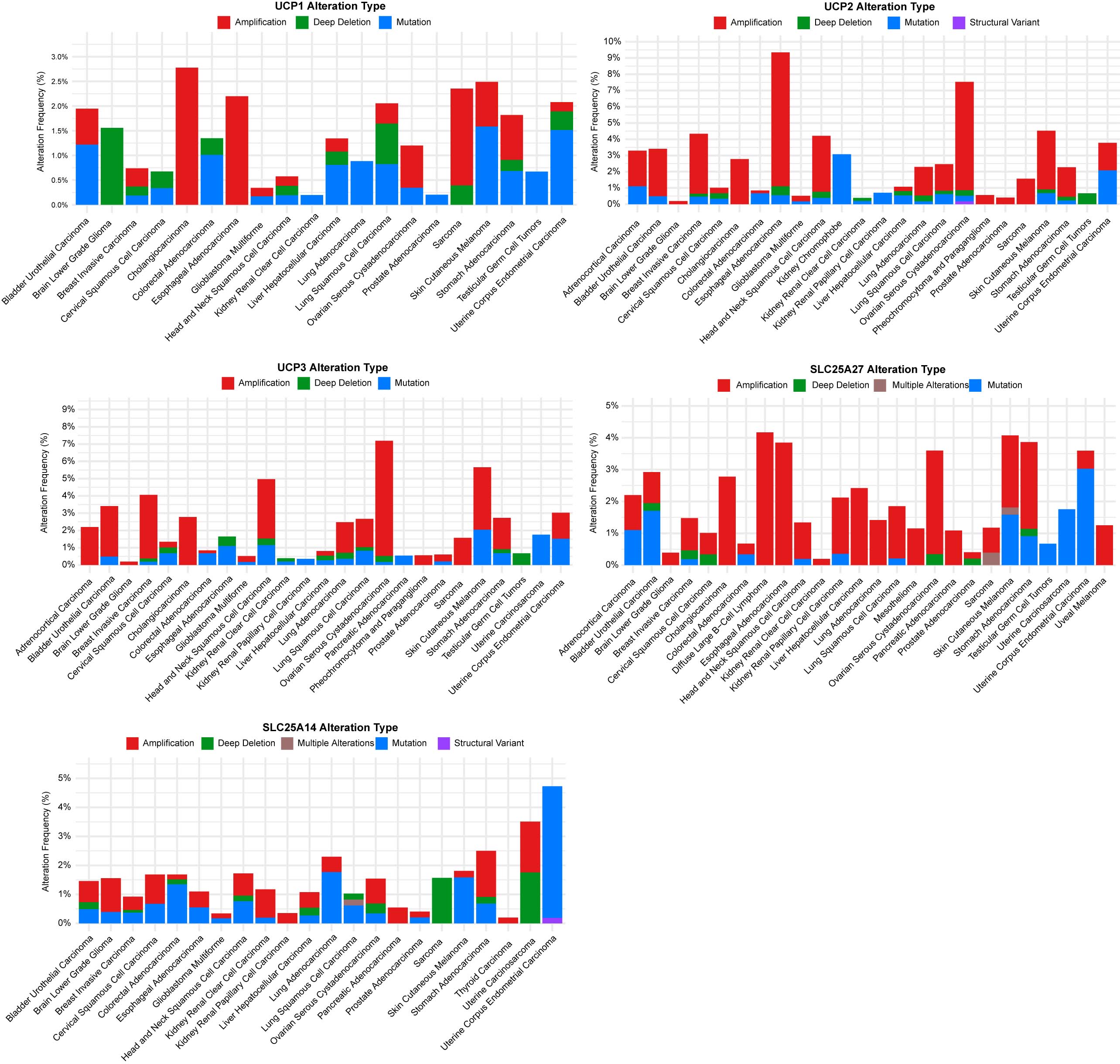


**FIGURE S2**

Frequency and genetic variations of each gene in the UCP family according to the cBioPortal database—pan-cancer analysis of whole genomes based on TCGA pancancer atlas.


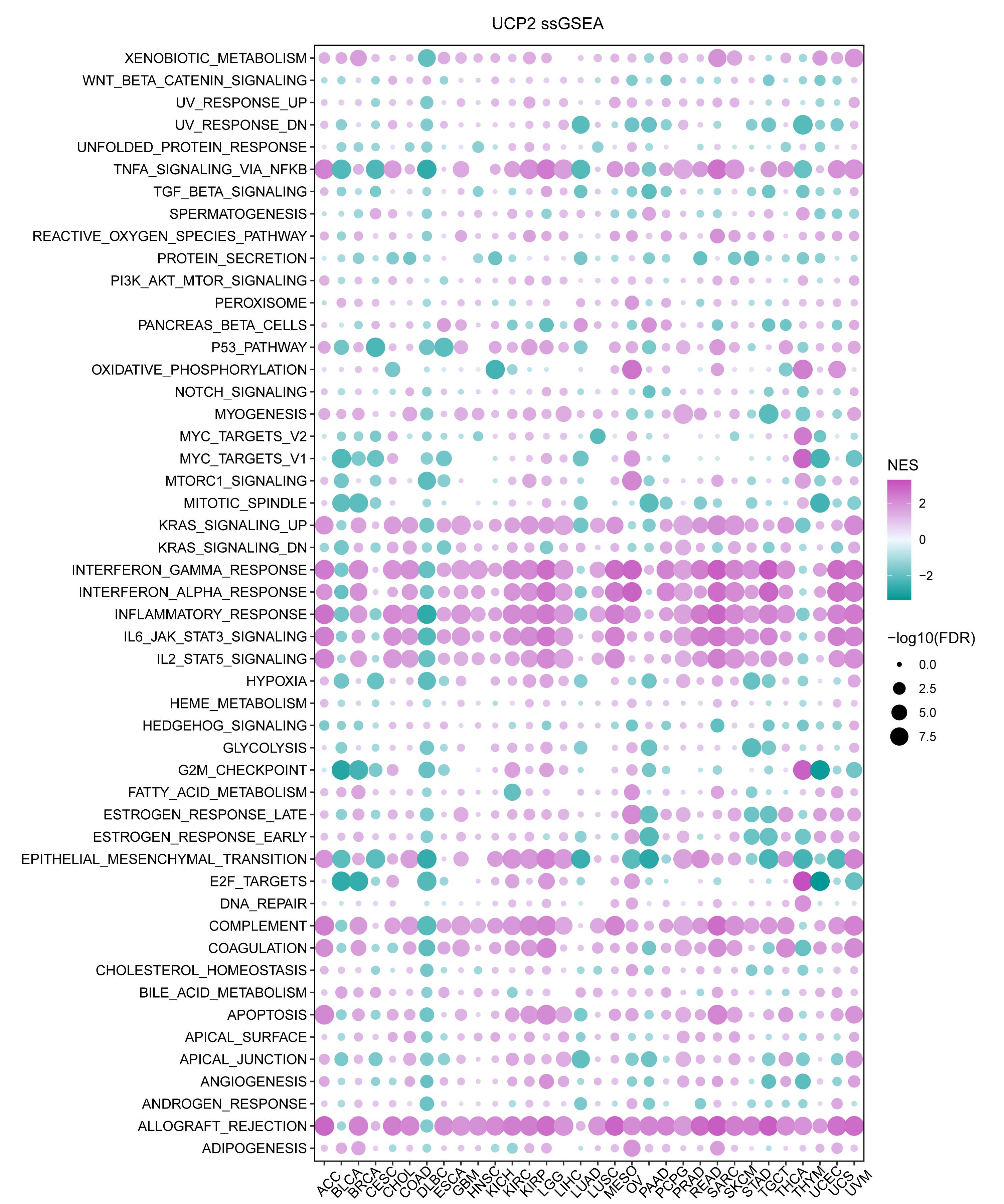


**FIGURE S3**

The hallmarks pathways of UCP2 in pan-cancer. The size of the circle represents the FDR value, and the color represents the NES.
